# The protocol of a population-based prospective cohort study in southwest of Iran to analyze common non-communicable diseases: Shahrekord cohort study

**DOI:** 10.1186/s12889-018-5364-2

**Published:** 2018-05-25

**Authors:** Arsalan Khaledifar, Morteza Hashemzadeh, Kamal Solati, Hosseion Poustchi, Valentina Bollati, Ali Ahmadi, Soleiman Kheiri, Keihan Ghatreh samani, Mehdi Banitalebi, Morteza Sedehi, Reza Malekzadeh

**Affiliations:** 1 Modeling in Health Research Center and School of Public Health, Department of Epidemiology and Biostatistics, Shahrekord University of Medical Sciences, Shahrekord, Iran; 2 Cellular and Molecular Research Center, Shahrekord University of Medical Sciences, Shahrekord, Iran; 3 Digestive Disease Research Center, Digestive Diseases Research Institute, Tehran University of Medical Sciences, Tehran, Iran; 4 Center of Molecular and Genetic Epidemiology, Department of Clinical Sciences and Community Health, Università degli Studi di Milano, Milan, Italy; 5 Clinical Biochemistry Research Center, Shahrekord University of Medical Sciences, Shahrekord, Iran

**Keywords:** Non-communicable diseases, Cardiovascular disease, Risk factors, Cohort study, Adults, Developing countries, Iran, Shahrekord

## Abstract

**Background:**

Prospective cohort studies are considered ideal choices to study multiple outcomes and risk factors for Non-communicable diseases (NCDs). Our aim is to set-up the protocol and analyze risk factors, incidence rates, prevalence, trends, and the models of environmental and genetic determinants of NCDs and their outcomes as well as interaction among such determinants.

**Methods:**

Shahrekord cohort study (SCS) that is a population-based prospective, study on a cohort consisting of people aged 35-70 years started in November 2015 in Iran. The sample size of the original cohort is at least 10,000 people. Annual follow-ups (200,000 person-year) of the cohort were designed to be conducted up to 2036. Exposures (a detailed demographic, socioeconomic, general health, quality of life, physical activity, anthropometric indexes, stress, health literacy, social capital, nutrition and eating habits, lifestyle, occupational history, living place, blindness, deafness, electrocardiography, lung capacities, blood pressure, sleep, smoking and alcohol, contact to animals, physical examinations and medical history, dental health, used drugs and supplements, glucose and lipid profiles) were measured by relevant standard methods and questionnaires. Incidence of common NCDs (cardiovascular diseases, cancer, gastrointestinal, respiratory, renal, hepatic, accidents, injury and neurological diseases), trend of risk factors, hospitalization, disability, and death were considered the outcomes of the cohort. The definition of disease was determined based on the International Classification of Diseases 10th version (ICD-10). Routine hematologic and biochemical tests were conducted and an all-inclusive biobank (blood, hair, nail, and urine specimens) of the cohort was stored for future studies. All steps of data collection and examinations are directly monitored by the quality control team.

**Discussion:**

The SCS is a unique study conducted in southwest of Iran that is a notable work given the climate conditions and ethnicity population (especially in Bakhtiari) of this region. By providing high quality the protocol and introduce it, the SCS can serve as a solid foundation for management and researchers in southwest of Iran. The SCS provides prerequisites for collaboration and regional, national, and international studies on NCDs. Data are available at the modeling in health research center, Shahrekord University of Medical Sciences, Shahrekord, Iran, for any collaboration.

## Background

In the recent years, policymaking, planning, and decision-making in public health and health system have entered a new phase of evolution that is widely known as evidence-based decision-making [1]. Adopting this approach is more essential to manage, care, prevent, and control non-communicable diseases (NCDs) than other diseases [2, 3], because according to the World Health Organization (WHO) projections, mortality from NCDs will increase by 77% between 1990 and 2020 such that seven out of every 10 deaths will be due to NCDs; most of such deaths are predicted to occur in developing countries and due to cardiovascular disease [4, 5]. Through the third epidemiological transition in the late twentieth century, NCDs and associated risk factors have become the most important health issue in many countries including Iran [6–10]. This issue is attributed to the paradox of human-machine (too much mechanized humans and too much humanized machines), increased median age of population, demographic changes and increased aging, the rapid growth of urbanization and globalization, smoking, high prevalence of exposure to risk factors for chronic diseases, genetic, improper diet, small communities, developing technologies and the subsequent decline in physical activities and increase in psychological-mental tensions [11–15].

In addition to affecting mortality rate, chronic diseases cause inability and influence productivity. Chronic diseases are associated with high prevalence, incidence, and disability-adjusted life year (DALY) in Iran, with various patterns in different provinces, as with other countries [14–18]. For example, the prevalence of multiple sclerosis (MS) is 830 people (92.71 per 100,000 population) in Chaharmahal and Bakhtiari (Ch&B) province (the setting of the current cohort study), representing the second leading prevalence of MS after Isfahan (93.06 per 100,000 population) in Iran [18]. It is therefore essential to address and give priority to NCDs in studies. Cross-sectional and case-control studies, and clinical trials suffer from certain drawbacks to assess exposures and associated long-term effects as well as the causes of NCDs’ outcomes, and therefore cannot be used to study these diseases closely. Prospective cohort studies can therefore be an ideal choice to investigate NCDs’ multiple outcomes, and various and concurrent risk factors as well as to develop the models of their environmental and genetic determinants and interaction among them [14–22].

## Methods/design

### The significance, aims and rationale of the SCS

It is widely acknowledged that smoking, obesity, increased intake of salt, and inappropriate lifestyle have adverse effects on health in Ch&B province, other provinces of Iran, and even other countries [6, 23–28]. However, the findings of studies conducted in European countries and the Americas and even Tehran and other metropolitans may not be applicable and generalizable to other regions of Iran such as Ch&B. For example, the prevalence of MS is seven times higher in Ch&B province than in north Iran (12.9 per 100,000 population) [18]; therefore, it can be argued that although MS is not a main health issue in north Iran, it can be considered a main health challenge in southwest of Iran particularly among active workforce. Other challenges in this province, compared to another province in Iran, are high prevalence of smoking, hypertension, tooth Decay/Missing/Filled (DMF), and lower happiness and social capital. On the other hand, in this province, with increasing trend of cancers, some cancers have high incidence than the expected rate compared to other cancer types including breast, bladder and gall bladder [19] for unknown reasons. It is therefore highly necessary to closely study and determine the NCDs’ load and control, genetic homogeneity in Ch&B province as people from comparatively more diverse ethnicities live in this province [29], its unique environmental and geographical conditions, and sociocultural diversity (especially in Bakhtiari) in southwest of Iran [30] as well as to investigate associated environmental and genetic factors and interaction among them. Besides that, we need to conduct specific cohort studies and make decisions based on local evidence to track, explain, and determine the trend of specific risk factors for NCDs and associated mortality, contributions of different exposures and general and specific causes of mortality in this province, stimulate policymakers and planners to promote the community’s health, apply knowledge and research findings to deliver healthcare services to people, help to promote the quality of life. Plan for moving towards precision medicine, capacitate and create high-quality scientific and research infrastructures in the only medical university of the province, and make arrangements for interaction with universities in Iran and abroad, and create a quality biobank in the province.

### Study type

The SCS is a population-based prospective cohort study with a 20-year follow-up. This study was designed to serve as one of the centers of the Prospective Epidemiological Research Studies in IrAN (PERSIAN) Cohort and is being conducted in Ch&B province, southwest of Iran [30–32]. The PERSIAN Cohort is a multicenter cohort study that was launched by the Deputy of Research and Technology of Iran Ministry of Health and Medical Education in 14 provinces. Further information is available at http://persiancohort.com [31].

### Setting and population

Ch&B province is located in Iran in central part of Zagros Mountain Chains and With 16.421 km^2^ area, this province comprises 1% of Iran’s total area and is the 22nd largest province of this country. According to report from Iranian statistics center (Iranian National Population and Housing Census) in 2016, the population of this province is 947763people (with sex ratio of 104 males/100 females). Ch&B province is one of the mountainous regions of Iranian Central Plateu, and is located between 31° 9′ to 32° 38′ north latitude and 49° 30′ to 51° 26′ eastlongitude GMT. Because of its mountainous nature and high altitude and located in the direction of Mediterranean systems moist winds that cause the rise and drainage of these systems’ loads, this province has relatively good rainfall. According to the latest national divisions, this province has nine counties consisting of Shahrekord, Ardal, Boroujen, Ben, Saman, Farsan, Kouhrang, Kiar and Lordegan. In this province, precipitation in the heights is mostly snow and snowy highlands represent one of the climatic specifications. Ch&B province neighbors Isfahan province from north and east, Khuzestan province from west, Lorestan province from northwest, and Kohgilooyeh and Boyer-Ahmad province from south. The population of this province are from Bakhtiari, Fars, Qashqai and Turkic ethnicities. Shahrekord, the capital of this province (with 315,980-individual population), is the most elevated provincial capital in Iran with 2066 m height above sea level. Ch&B province’s height above mean sea level is around 2153 m and therefore this province is known as Iran’s roof [32].

### Adequate sample size

The required sample size was calculated by three Kelsey, Fleiss or Fleiss with continuity correction formula, with Two-sided significance level (1-alpha) 95%, 0.85% Power (1-beta, % chance of detecting), and unexposed/exposed ratio of 1, 5% of unexposed with outcome and risk/prevalence ratio or odds ratio 1.3, we need 4899 exposed group, 4899 non-exposed group and therefore total sample size 9798. Required sample size using Fleiss or Fleiss with continuity correction formula was derived, respectively, 9794 and 10,078 person [33]. Considering 2-5% nonresponse, attrition rate or follow-up loss, decrease in random error and target population census in two geographical areas, we decided to enroll 10,078 persons (200,000 person - Year of follow up).

### The selection of the participants: Target region, population and sampling

The cohort was chosen from people aged 35-70 years in studied regions by census.

**Fig. 1:**
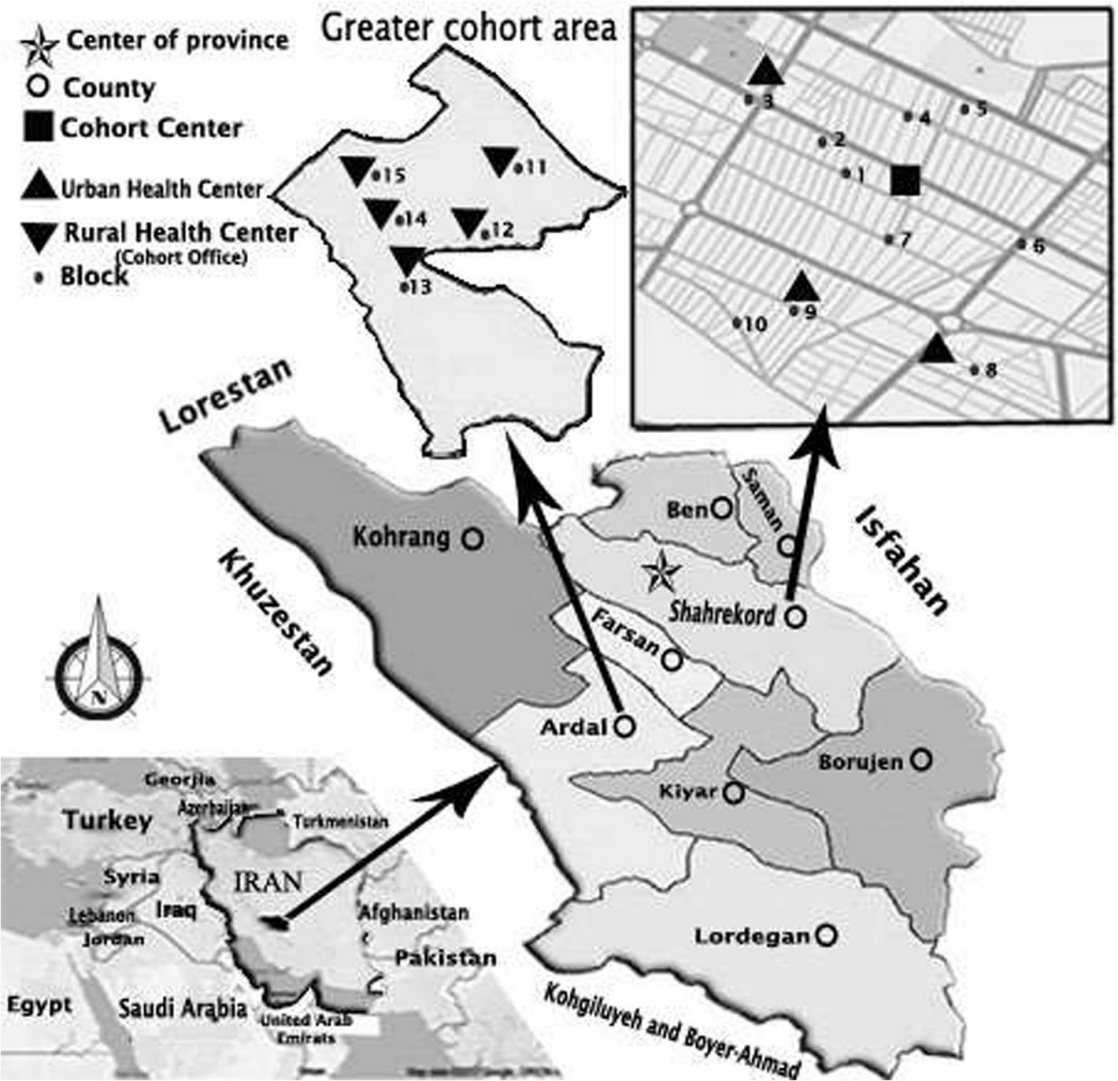
The map of Iran, the location of Chaharmahal and Bakhtiari province, and Shahrekord Cohort Study. Legend:(Source: Organization of Management and Planning of Chaharmahal & Bakhtiari Province, National Mapping Organization)

Study regions in the SCS were selected by a multistage, stratified cluster random sampling method. The SCS sample consists of two stratified clusters (urban and rural). Numbers were assigned to the blocks (as clusters) of each stratum and then selected participants by census. We invited all eligible adults residing in each block selected and then moved to the next block, repeating the invitation process, until we gained the sample required for that stratum. Sample size for each stratum was determined proportional to the number of population in each stratum. Around 65-70% of this province’s population lives in urban areas and the rest do in rural areas. Shahrekord cohort center (urban cohort) and five office fields (rural cohort) were funded in the selected regions to facilitate the selection of the participants. The geographical map of the Cohort, neighbors and offices are shown in Fig. 1.

**Fig. 2:**
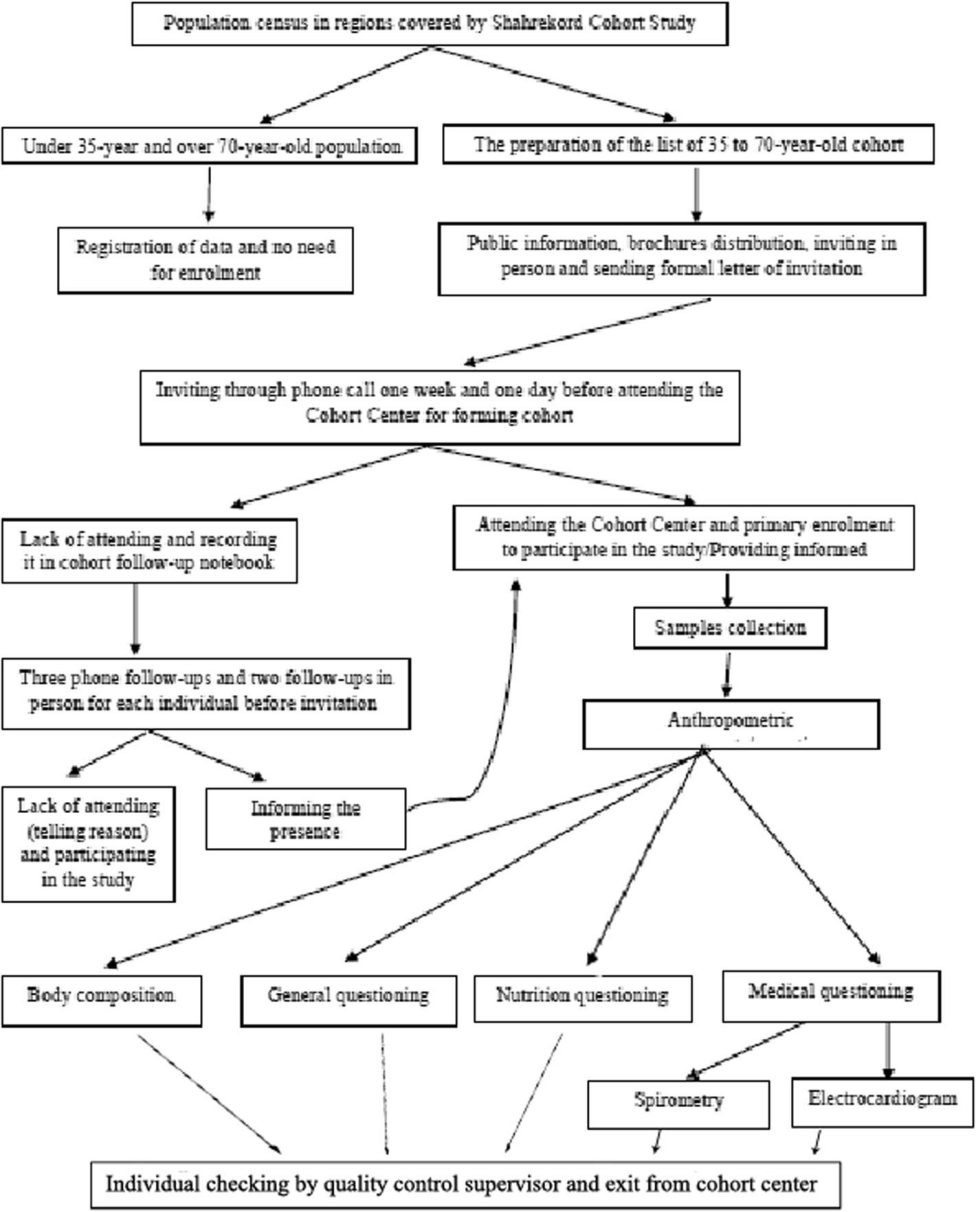
The cycle of the participants’ referring to the Cohort Center

To select these regions and sample this cohort, several meetings were attended by the provincial healthcare system officials and a number of scientific committees were formed by the faculty members of epidemiology and biostatistics as well as those of clinical departments of the Shahrekord University of Medical Sciences (SKUMS). Besides that, an Experts Panel was convened and certain factors such as cluster sampling, the samples’ accessibility and collaboration, the samples’ representativeness to maximize the generalizability of the findings to the entire province, low rate of migration, virgin and distinct ethnicities from other regions of Iran, and the availability of facilities needed to conduct longitudinal studies were taken into account to select the regions of interest. To collect primary demographic information on participants from Shahrekord urban areas, we first drew the map of these regions and then subdivided it into different blocks. Afterwards, data on people covered by the cohort map blocks were compared to those registered in the Integrated Health System (SIB network) of the healthcare service delivery centers by interviewers who had undergone training courses on census, and then a list of people living in the studied regions was prepared and finalized. Demographic data on people from rural areas were collected and finalized in the Ardal Healthcare Network, using the census list of rural health house workers (*Behvarz*) and with assistance of healthcare workers that are mostly native to the region where they work and therefore have sufficient information about the covered population. Information about the study and its protocol was and is still being publicized using brochures and mass media. The cohort samples are recruited by asking them to attend the Cohort Center and participate in the SCS at census when they referred and by telephone call two weeks before attending the Cohort Center. To invite people to participate in the SCS, a dated letter of invitation was first given to them in person, and then, for the second and third time, they were reminded by telephone respectively one week and one day before the day of their referral. The cycle of the participants’ referring to the Cohort Center is shown in Fig. 2.

Eligibility criteria consists of both sexes, aged 35-70 years, only residing in the limited geographic cohort and for at least 1 year prior to the recruitment, no plan to leave Shahrekord for the following 1 years after the recruitment, adequate physical and mental ability to participate in the evaluation program and signing of written informed consent. Exclusion criteria consist of lack of residing in cohort geographical area and unwillingness to participate in the study.

### The steps and phases of the SCS

A) Step 1. Phases of preparation, quality assurance and quality control (pre-pilot, and pilot of the cohort): First, all necessary equipment, building, exclusive laboratory, bio-bank with seven refrigerators, monitoring temperature system, uninterruptible power supply(UPS), standard questionnaires, field and sources were prepared based on the PERSIAN Cohort Protocol [30, 31] and Shahrekord cohort proposal [32]. Then, consumable and non-consumable equipment and instruments, physical space, and human sources were monitored and evaluated using a checklist, and internal and external observers were qualified. The pre-pilot and pilot steps of the SCS were conducted in the covered regions within 12 months, from late November 2015 to late December 2016.

### SCS sources

A field team is working every day in the cohort office. The team consists of physicians, interviewers, nurses, sampling technicians, manager, supervisor, driver and office boy. The questionnaires are administered and completed by trained interviewers with at least master’s or bachelor’s degree. All workers have been selected from 150 applicants after a primary test and interview. The interviewers attended the SCS training courses (two theoretical and practical 6-day workshops), hold a reliable certificate issued by Iran Ministry of Health and Medical Education, and were contracted exclusively for the SCS by a private company that signed a contract with the SKUMS. Besides that, in-service and monthly training courses were held for the interviewers. The SCS is guided by a chief executor and a main collaborative-executive team consisting of geneticist, epidemiologist, biostatistician, psychologist, and cardiologist as well as a technical and scientific advisory team consisting of nutrition specialist, gastroenterologist, internist, pulmonologist, nephrologist, medical informatics specialist, medical geneticist, oncologist, radiologist, orthopedician, molecular medicine specialist, gerontologist, health expert, pharmacologist, neurologist, nurse, and ophthalmologist. In addition, an executive team was set up in a building with about 350 m^2^ of area, interview stations with 23 employees, a laboratory, and a biobank collectively with about 150 m^2^ of area to register exposures and collect the data of the cohort. The staff of the SCS consist of a receptionist, a field moderator, three medical interviewers, three general interviewers, one individual in charge of anthropometrics, five nutrition interviewers, three laboratory and sampling technicians, one technician in charge of the Central Cohort Biobank, two individual in charge of the Cohort Center quality control, two individual in charge of the follow-up for cohort and the chief of the Cohort Center (an epidemiologist). A general practitioner and a cardiologist attended the Cohort Center roughly every day at predetermined hours, and supervised and managed all measures taken to invite, register, and interview with the participants, sampling and laboratory activities, quality control, and the follow-ups.

B) Step 2. Conducting the main phases of the SCS from late September 2015 to 20 years later (2036).

**Enrolment phase:** At this phase, all samples are gradually enrolled by protocol. After the participants are registered and filled out informed consent forms, the exposures are measured by relevant standard tools and questionnaires. Data on general health, personal and working life history, medical examination, the frequency, prevalence, and history of NCDs, exposure to and risk factors for NCDs, lifestyle, physical activity, and nutrition (Food Frequency Questionnaire (FFQ)) are collected by electronic questionnaires. Tools used in the Shahrekord Cohort Study were shown in Table 1.

**Table 1:** Tools used in the Shahrekord Cohort Study

Dimension/questionnaires/check list	items	Assessment in
General	157	Whole cohort
Nutrition	153	Whole cohort
Medical history and examination	185	Whole cohort
General Health (GHQ12)	12	Whole cohort
Quality of life (WHO-QOL)	21	Whole cohort
Modified WHO MONICA	50	Whole cohort
Chronic stressor	46	Whole cohort
Coping strategies	31	Whole cohort
Pattern and smoking causes	132	Subgroup
Oswestry low back pain	10	Subgroup
Social capital and happiness	44	Subgroup
COPCORD^a^	100	Subgroup
Health literacy	33	Subgroup

^a^Community oriented program for control of rheumatic diseases

At this phase, blood, urine, hair, and nail specimens are gathered and stored in the SCS Biobank. Division and isolation of blood and urine samples for every individual in the Shahrekord Cohort Biobank were shown in Table 2.

**Table 2:** Division and isolation of blood and urine samples for every individual

The type of VENOJECT® tube	Aliquots type	Aliquots number	Temperature (°C) storage in Biobank
clot	serum	2	−80
EDTA (with k3)	Whole blood	3	−80
	plasma	7	−80
	Buffy coat	3	-80
urine	urine	1	−20
total		16	–

### Phase 2: Follow-up and outcomes ascertainment

The follow-ups of the cohort were decided to be conducted once a year by phone. If there was no answer to phone calls after six times in three different days of three weeks, the follow-up team will go to the home of the participant and interview him/her face to face. For rural cohort, Behvarz will facilitate this process. If outcome occurs, the participants are visited in person. Meanwhile, the cohort is tracked by referring to the participants’ medical records, hospital files, cancer registry and different registries including the SIB network and hospital information system (Samaneh Electronici PArvandeh Salamat-SEPAS) to assess their conditions.

The incidence rates of the outcomes in question including death and NCDs (cardiovascular and gastrointestinal diseases, cancers, diabetes, pulmonary diseases, crashes and accidents, and neurological diseases), trend of risk factors, hospital stay, and disability are followed up and measured with two types of active or passive follow-up. The definition of disease was determined based on the International Classification of Diseases 10th version (ICD-10). The death will be registered and if the other outcomes of interest occur, the participants will be invited to the laboratory to collect their blood samples. In the case of a death report but no reliable death certificate, a verbal autopsy will be performed to determine cause of death. Outcomes will be extracted from SEPAS and SIB using the national identifier number of each subject to link data. The national identifier number is a unique identifier of Iranians used in national databases. In addition, they are thoroughly checked up and periodically examined once every 5 years in 5, 10, 15 and 20th years of follow-up. The follow-up team, The Outcome Ascertainment Committee and the SCS Executors (PIs) participate in this step. Three to five years after enrolment, the health status is reassessed in sub-cohort and primary examinations and the questionnaires administration are duplicated in the light of current epidemiological conditions, available evidence of cross-sectional studies, the ongoing trend of risk factors and different studies, particularly epigenetic investigations (studies on common biomarkers in prepared specimens in the outcomes) are conducted [14].

I) The quantification of specific molecules (e.g. DNA or RNA adducts) and early biological markers (e.g. somatic mutations), II) the better characterization of study subjects (e.g. genomic variations in metabolic genes) and their risk stratification, and III) the use of molecular markers to further classify diseases that are currently categorized by etiology or prognosis. In addition, a number of nested case control, case-cohort, clinical trials, and cross-sectional studies will be specifically conducted. The contributions of associated risk factors for exposure to NCDs, genetic and environmental factors and interaction among them will be determined. Arrangements will be determined to closely investigate the trend of changes in the prevalence and incidence rates of risk factors for NCDs (hyperlipidemia, hypertension, diabetes, obesity, etc.) and life expectancy through the phases of the SCS. Besides that, data on mortality rate for different reasons (e.g. myocardial infarction, stroke, cancers, and MS) will be collected during the SCS and through additional studies.

### Instruments of data collection, exposures and specimens

After primary enrollment of the people at referral and completion of the informed consent form, each individual is assigned an 11-digit code namely PERSIAN Cohort Identification (PCID). The first four digits represent the province and city where the code holder lives, the following digit does the living place conditions consisting of city, main village, satellite village, and mobile village, the following four digits do family positions that can be householder, spouse, child, grandmother, and grandfather. Main questionnaire used to collect the data was derived from the PERSIAN Cohort Web-based Electronic Standard Questionnaires [31] that have already been nativized. These questionnaires were reported to have acceptable levels of validity and reliability [16, 34–37] as follows:

1. General Questionnaire: To collect individual and household demographic and socioeconomic information, occupational history and exposures, the status of fuel and living place, lifestyle, the type of used water, housing, sleep status, history of exposure to animals, pesticides, and cell phone, physical activity (in MET), quality of life, and general and social health;
2. Nutrition Questionnaire: To collect data on the frequency of used foods, supplement and eating habits;
3. Physical exam and medical Questionnaire: To investigate history of fertility (for women), history of chronic diseases, used drugs, family medical history, oral health, physical examination and disability, and individual habits (smoking and alcohol use). Blood pressure was measured twice (15-min interval) in the right and left arms using a standard barometer (Richter). Pulse rate was measured for 60 s twice with at least 5-min interval. The 12-lead electrocardiograms (EKG) and pulmonary function tests (spirometry) of all participants were taken and are stored in a standard format after automatic diagnoses by Cardiax® and Spirolab (MIR, Italy) software, and then the readings are examined, reviewed, encoded, and interpreted by a cardiologist and a pulmonologist.
4. In the Cohort Laboratory, a 25-ml blood sample, around 500 hairs 1 to 3 cm long, 1-mm nail, and around 15 to 25-mm urine samples were taken from each participant. The specimens are stored under ideal conditions and out of reach in the SCS Biobank. Some amount of the blood samples was used to conduct routine hematologic tests including complete blood count (CBC); (Nihon-koden cell counter) and biochemical tests (fasting blood sugar, serum total cholesterol, triglyceride, asparagine aminotransferase, alanine aminotransferase, high and low density lipoprotein, cholesterol, alkaline phosphatase, gamma glutamyl transpeptidase, vitamin D, thyroid stimulating hormone, urea and creatinine levels) and urine analysis. The results of the tests are recorded and delivered to the participants.
5. In the Anthropometrics Unit, the height was measured by a wall height meter (Seca 206), weight measured by an analog scale (Seca), and waist, hip, and wrist circumferences measured by a standard tape measure. Body components such as fat, water, and muscle were measured by a body composition measure (Tanita, Japan).

### The SCS quality control

Because high quality data are essentially required for epidemiological and cohort studies, quality control team in this study was convened at two levels: National [the PERSIAN Cohort], and university/executive. At university level, the members of the quality control team are faculty members of the departments of epidemiology and biostatistics, biochemistry, and laboratory sciences. To control field quality, this team also includes three people with master’s degree on epidemiology, biostatistics, and laboratory sciences. All steps of data collection such as census, invitation, examinations, interview, and sampling as well as taking tests and recording their results are directly monitored by the quality control team.

The participants complete the executive steps. A checklist is used to monitor the steps and the duration of measuring the exposures. If a participant does not complete any executive step, the reason is recorded and the participant required to have the relevant document till the completion of the executive steps so that he/she can receive the Cohort Identification Card. The checklists data are compared to those registered in the electronic questionnaire by the quality control team to remove potential inconsistencies. The accuracy of the completed items are examined with reference to the outputs of the electronic questionnaire that were converted to an MS Excel file. Every month, three interviews are conducted with each participant, as the interviewer is blind, and the interviews are recorded. The methods of the interviews and the accuracy of the collected data are examined. In some cases, the participants are randomly asked to refer to the Cohort Center again to recomplete a section of the questionnaire (In this step, anonymous paper questionnaires are used) to examine the consistency of the data drawn after each administration. Besides that, a central quality control team of the PERSIAN Cohort monitors affiliated studies regularly and meticulously according to a well-developed protocol. Field quality, the performance of the interviewers, and the calibration of the used equipment are assessed, monitored, and evaluated according to the processes of each part of the interviews and using a checklist daily, weekly, monthly, once every 3 months and annually and the results are recorded.

## Discussion

The SCS is a unique study conducted in southwest of Iran that is a notable work given the climate conditions and various population (especially in Bakhtiaris) of this region. Aimed to study chronic NCDs and associated factors, this study can, in the first place, serve as a solid foundation for planning and policymaking in healthcare field and the control of common diseases of the region, provinces of Iran and secondly, be useful for other countries of the Eastern Mediterranean and even worldwide. Although a number of cohort studies were or are being conducted in the Eastern Mediterranean, the current study, representing one of the centers of the PERSIAN Cohort, can provide highly valuable evidence on precision medicine. Notably, the main exposures are measured and registered in the SCS as with other centers of the PERSIAN Cohort. Although the SCS was started a little late following other centers of the PERSIAN Cohort, this delayed initiation became an opportunity as the executors could observe, visit, and gain experience with already initiated cohort studies. As a result, in the SCS, the PERSIAN Cohort Protocol is being thoroughly followed and, according to the codified protocol, certain variables that are likely to play confounding role in analyzing associations and outcomes or be colliders are measured and registered as well. Therefore, an advantage of the SCS over other centers of the PERSIAN Cohort, is taking the EKG, pulmonary function test, and pulse oximetry, measuring body composition, general health, social health, chronic stressor, coping strategies, health literacy, and low back pain as well as conducting examinations for rheumatologic disorders using valid and nativized questionnaires. SCS provides useful evidence for research in social epidemiology, NCDs epidemiology, health services research and genetic epidemiology. In certain centers of the PERSIAN Cohort, a number of these variables are studied. For example, in the Yazd Cohort Study, spirometry is taken, in the Azar Cohort Study, rheumatic diseases are investigated [38], and in Fasa Cohort Study, body composition is measured and EKGs are taken [39]. Enrolling people from both urban and rural areas is another strength of the SCS that can help us achieve highly valuable evidence on different aspects of NCDs’ etiologies and factors associated with living in rural and urban areas. In the SCS, demographic information is collected and finalized in urban and rural areas, respectively, using the SIB network and with assistance of health workers (Behvarz) that is highly important for the following steps of the SCS including the cohort outcomes ascertainment and long-term follow-up, because reliable evidence can be achieved in cohort studies if participation rate remains high not only at the initial steps but also through years of follow-ups. Hiring an efficient quality control team to monitor the steps of data collection using checklist is another remarkable strength of the SCS. Controlling and ensuring quality represents a serious concern that is being widely taken into account, by the SCS executors, as a sensitive factor.

## Abbreviations

Ch&B: Chaharmahal and Bakhtiari
EKG: Electrocardiograms
FFQ: Food Frequency Questionnaire
NCDs: Non-communicable diseases
PCID: PERSIAN Cohort Identification
PERSIAN Cohort: Prospective Epidemiological Research Studies in IrAN
SCS: Shahrekord cohort study
SEPAS: Samaneh Electronici PArvandeh Salamat
SKUMS: Shahrekord University of Medical Sciences
WHO: World Health Organization
